# Effect of Applied Pressure on the Electrical Resistance of Carbon Nanotube Fibers

**DOI:** 10.3390/ma14092106

**Published:** 2021-04-21

**Authors:** Chris J. Barnett, James D. McGettrick, Varun Shenoy Gangoli, Ewa Kazimierska, Alvin Orbaek White, Andrew R. Barron

**Affiliations:** 1 Energy Safety Research Institute, Swansea University Bay Campus, Swansea SA1 8EN, UK; c.j.barnett@swansea.ac.uk (C.J.B.); v.s.gangoli@swansea.ac.uk (V.S.G.); ewa.kazimierska@swansea.ac.uk (E.K.); alvin.orbaekwhite@swansea.ac.uk (A.O.W.); 2 SPECIFIC, Swansea University Bay Campus, Swansea SA1 8EN, UK; j.d.mcgettrick@swansea.ac.uk; 3 Department of Chemistry and Department of Materials Science and Nanoengineering, Rice University, Houston, TX 77005, USA; 4 Faculty of Engineering, Universiti Teknologi Brunei, Jalan Tungku Link, Gadong BE1410, Brunei

**Keywords:** carbon nanotubes, fiber, pressure, XPS, conduction

## Abstract

Carbon nanotubes (CNTs) can be spun into fibers as potential lightweight replacements for copper in electrical current transmission since lightweight CNT fibers weigh <1/6th that of an equivalently dimensioned copper wire. Experimentally, it has been shown that the electrical resistance of CNT fibers increases with longitudinal strain; however, although fibers may be under radial strain when they are compressed during crimping at contacts for use in electrical current transport, there has been no study of this relationship. Herein, we apply radial stress at the contact to a CNT fiber on both the nano- and macro-scale and measure the changes in fiber and contact resistance. We observed an *increase* in resistance with increasing pressure on the nanoscale as well as initially on the macro scale, which we attribute to the decreasing of axial CNT^…^CNT contacts. On the macro scale, the resistance then *decreases* with increased pressure, which we attribute to improved radial contact due to the closing of voids within the fiber bundle. X-ray photoelectron spectroscopy (XPS) and UV photoelectron spectroscopy (UPS) show that applied pressure on the fiber can damage the π–π bonding, which could also contribute to the increased resistance. As such, care must be taken when applying radial strain on CNT fibers in applications, including crimping for electrical contacts, lest they operate in an unfavorable regime with worse electrical performance.

## 1. Introduction

Copper and aluminum are the industry standards for electrical conductors, and which one is used depends on the application. Where weight is an issue, aluminum provides a better solution because it is lighter than copper; however, when more power density is needed, the higher conductivity (and thus smaller size) of a copper cable is selected. Unfortunately, when considering large scale power transmission, ca. 10% is lost, primarily due to resistive heating effects within the cables. To compensate for each 200 MW of line loss, the equivalent of a coal plant must be online with the associated carbon emissions. In addition to issues associated with power loss, the weight of any conductor has a significant impact on energy consumption. This is particularly true in the automotive and aerospace industries [1].

The all-carbon nanotube wire, in which the carbon nanotubes (CNTs) are all metallic in nature, was first proposed by researchers at Rice University [2]; however, while commercial CNT fibers are manufactured, they are composed of a mixture of metallic and semiconducting CNTs [3,4,5]. These CNT fibers can be spun using CNTs manufactured using various methods, which results in the production of fibers that have different qualities [6,7]. Drawing and spinning arrays from forests of CNTs can result in fibers with high tensile strengths; however, the CNT lengths are variable and limited by the size of the vertical array [8]. Another method involves drawing CNTs directly from a chemical vapor deposition (CVD) furnace [9,10]. It has been reported that CNT fibers have high conductivities on the order of MS/m and are comparable in terms of specific conductivity to metals [11,12]. However, irrespective of the method of manufacture, it has been found that the purity of the resulting fibers is commonly sub-optimal and hence their conductivity is never as good as it could be. 

During production, and when in use, CNT fibers can be under strain, especially in important applications in cables or windings. Work carried out by Wu et al. showed that this type of longitudinal strain along the length of the fiber can increase resistance [13,14], adding another layer of complexity when considering the optimum possible conductivity of a CNT fiber. However, one area not previously considered is that when they are used for electrical and signal transport in many applications, fibers may undergo crimping, which puts compressed radial strain across the diameter of the fiber. Herein, we investigate how this type of strain will affect the electrical resistance on both the nano- and macro-scale by applying radial compressive pressure through nanoscale and macro-scale contacts on Nanocomp’s retail single-ply YE-A10 carbon nanotube fiber while measuring the corresponding resistance changes. We also use X-ray photoelectron spectroscopy (XPS) and UV photoelectron spectroscopy (UPS) to understand whether applying pressure on the fiber changes the electronic structure of the nanotubes.

## 2. Materials and Methods

### 2.1. Materials

A 100-m yarn of YE-A10 carbon nanotube (CNT) fiber was purchased from Nanocomp Technologies, Inc. (Merrimack, NH, USA) and handled as described before [15]. This is a single-ply fiber of single- and few-walled CNTs with a rated average diameter of 130 µm. A 60 cm length of Nanocomp YE-A10 carbon nanotube fiber was placed in a 20 mm diameter quartz tube and annealed in a Thermo Scientific (now part of Thermo Fisher Scientific, Waltham, MA, USA) 545000 high temperature tube furnace operating at a base pressure of 1 × 10^−3^ mbar at 400 °C for one hour, and was then allowed to cool under vacuum. This was done to remove surface contamination that could result in inconsistent contacts to the fiber [16]. The annealed fiber was cut into a 30 cm piece, a 1 cm piece and two 10 cm pieces.

### 2.2. Nanoscale Measurements

**Figure 1:**
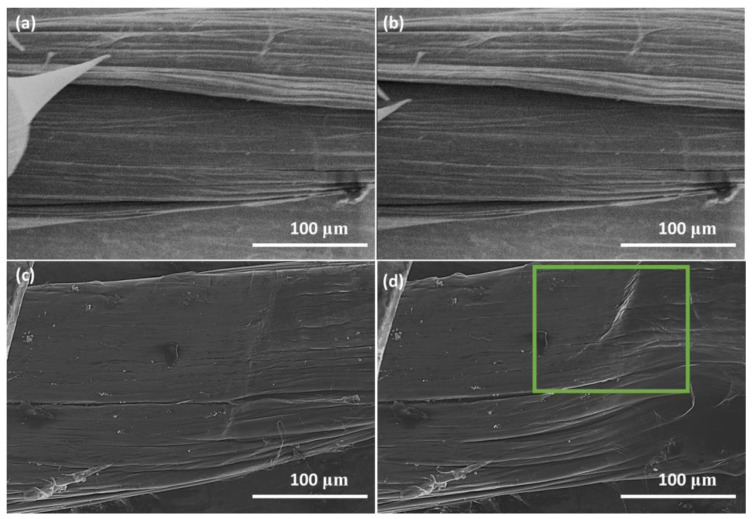
SEM images of carbon nanotube fiber (**a**) with nanoscale probes on twist, (**b**) nanoscale probe removed after measurements, (**c**) before macroscale measurements and (**d**) after macroscale measurement showing compression from radial strain showing crimping inside the green box.

The 1-cm long piece of annealed fiber was placed inside an Omicron LT Nanoprobe (now part of Scienta Omicron, Uppsala, Sweden) operating at a base pressure of 1 × 10^−10^ mbar. Two tungsten scanning tunneling microscope (STM) probes, that had previously been annealed to remove shank oxide to ensure consistent contact [17,18], were introduced to the surface, along a twist of the fiber (Figure 1a) at varying tip separation distances using a previously described method to ensure no strain-induced conduction changes [19]. When the tips were in contact with the fiber, as measured by the real time current sensors, five separate current measurements were taken for a voltage sweep from −1 V to +1 V for statistical accuracy, and at different points along the fiber surface to account for any material inconsistency. One of the probes was then lowered further using the STM system and pressed into the sample in increments of 100 nm to induce radial strain, and the entire system of measurements as described above were conducted at each such increment.

### 2.3. Macroscale Measurements

**Figure 2:**
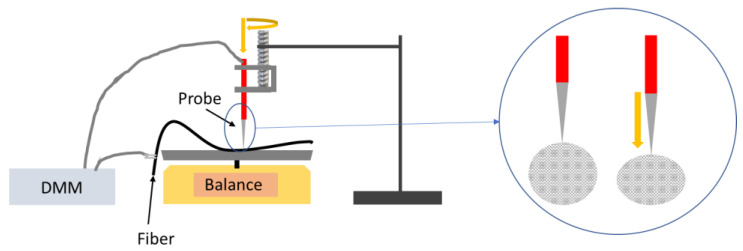
Diagram of macroscale measurement setup to induce radial strain and cross section of tip inducing strain on the carbon nanotube fiber.

The 30-cm long fiber section was placed on an Ohaus Scout Pro balance (Scout Pro Portable Balance, Ohaus, Switzerland) with a single wrap-around banana-style Mueller Electric Digital Multimeter (DMM) test lead to act as a contact without any applied pressure. A second contact was made using a second such test lead that was attached to a screw so the rod could be lowered onto the fiber surface to induce radial strain and so that the pressure applied on the fiber could be measured in terms of applied effective mass using the weighing balance. A diagram of the experimental setup is shown in Figure 2. The distance between the contacts was held constant at 25 cm, and a voltage of 5 V was applied across the fiber section between these contacts, with the current from the applied potential measured. Between each measurement, the test lead was brought out of contact with the fiber and then re-approached for the next measurement.

### 2.4. X-ray Photoelectron Spectroscopy

One of the two 10-cm long pieces of annealed fiber was placed inside a Chicago Pneumatic CP86150 press (Chicago Pneumatic, Chicago, IL, USA) between two CNC-machined ultra-flat aluminum disks and placed under 3 US-tons of pressure, thrice, for 30 s each time. Any higher and the fiber was susceptible to irreversible damage to the structural integrity. This piece, as well as the non-pressed piece (control), was characterized in a Kratos Axis Supra XPS (Axis Supra, Kratos, Manchester, UK) at a base pressure of ~1 × 10^−9^ bar. As the typical analysis area of this system is approximately 300 × 700 µm, the fibers were mounted free-standing over the relatively large (cm scale) holes on the sample bar to remove any risk of substrate influence in the final data, with each point at least 2 mm apart. The conductive fibers were mounted in an electrical connection with the ground. As such, the charge neutralizer was not used, and no charge correction was applied to the data. X-ray photoelectron spectroscopy (XPS) and ultraviolet photoelectron spectroscopy (UPS) spectra were collected for 6 separate sections of 700 µm each, per fiber, and analyzed for statistical accuracy. XPS was run using Al-K_α_ (15 mA, 225 W) emission with a pass energy of 40 eV and step size of 0.1 eV. UPS was run using He(I) emission using a 110 µm aperture, pass energy of 10 eV and a step size of 0.025 eV. Data were analyzed in CasaXPS (Version 2.3.23rev1.1K).

## 3. Results and Discussion

Conduction measurements were carried out along a twist of the annealed fiber (Figure 1a) in the Omicron LT Nanoprobe with probe spacings of 20 µm, 80 µm, and 220 µm, and the resistance was calculated from the average current measurements at 0.5 V. Our prior research has demonstrated that measurement over these probe separation distances obviates any detrimental effects of depletion zones around probe-CNT contact that make measurement unreliable [16]. For a simple conductor, it is typical that the resistance measurements increase with probe separation; however, there is no such trend observed here. The resistance at 20 µm was measured to be 3.17 × 10^4^ Ω while the resistance with a probe separation of 80 µm and 220 µm was calculated as 9.57 × 10^3^ Ω and 9.61 × 10^3^ Ω, respectively. This is due to two effects. First, the composition of the Nanocomp CNT fibers as received from the company is inhomogeneous due to the presence of amorphous carbon, voids, and iron catalyst residue as well as the twisted yarn structure [8,20]. Second, when the probe separation distance is close to or larger than the average fiber diameter itself (130 µm), the current pathway can go beyond being a surface phenomenon and through the entire diameter of the fiber.

**Figure 3:**
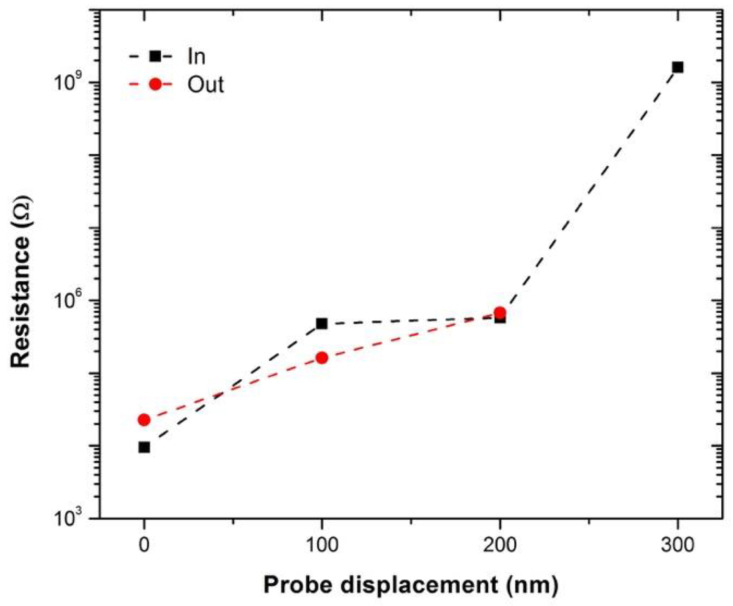
Graph of electrical resistance of a CNT fiber measured using 80 µm probe separation against STM probe displacement: black squares show probe approach and red circles show retraction.

**Figure 4:**
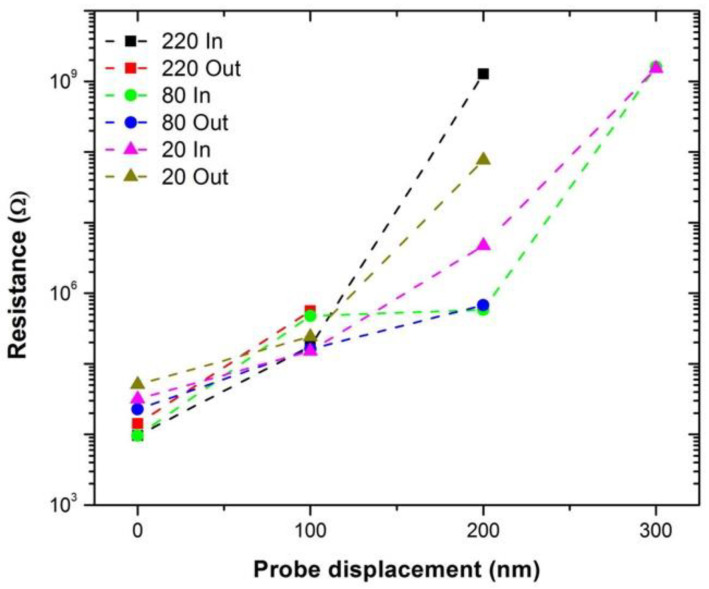
Graph of electrical resistance of a CNT fiber measured using different probe separations (20 µm (triangle), 80 µm (circle), and 220 µm (square)) against STM probe displacement approaching (in) and retracting (out) from the CNT fiber.

Figure 3 shows a plot of the 2-point probe measured electrical resistance at a probe separation of 80 µm as a function of one of the probes approaching and being withdrawn in 100 nm increments into the Nanocomp YE-A10 fiber. It should be noted that the measurements were taken with an STM system, and therefore we do not know the exact force exerted by the piezo material controlling the tip position, or the exact contact area under the probe [19]. Given this limitation, it is not feasible to accurately calculate the applied pressure and induced radial strain from the probe on the fiber, and therefore the results are presented in terms of extension of the piezo material (in nm). As the pressure is increased, the measured resistance also increases, and when the tip is withdrawn, the measured resistance was found to reduce. The same trend was observed for the two other probe separations as well, as shown in Figure 4 and repeat measurements of the 20 μm carried out on another part of the fiber as shown in the Supplementary Materials, Figure S1. It should be noted that the resistance measurement returns to a slightly higher value than originally measured, and this may be caused by the slightly displacement of the fibers on the sample holder, internal irreversible displacement of the fibrils, or induced strain on the system; however, these were not observable under SEM (Figure 1b).

**Figure 5:**
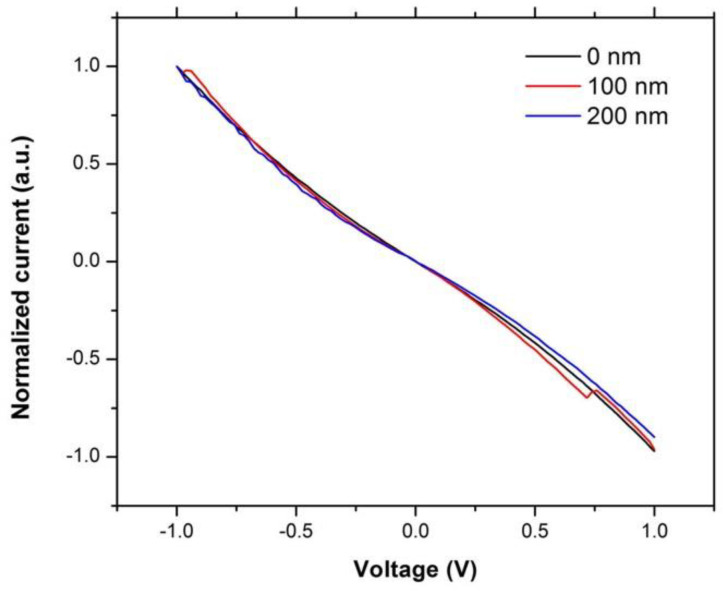
Representative I–V measurements of electrical resistance of CNT fiber measured using 80-µm probe separation against STM probe displacement of 0 nm, 100 nm, and 200 nm, cf., Figure 3.

For a fiber prepared from individual filaments (in this case CNTs) it would be expected that the electrical resistance would decrease with increased pressure due to the improved contact between the probe and the fiber as well as between the individual filaments [21]. Surprisingly, for CNT fiber, we have observed the opposite. Although it is known that strain can increase the measured resistance of a material due to lattice stretching [22], this is unlikely to cause a 6-order magnitude increase in electrical resistance that is observed here (cf., Figure 4). It is known that pressure-induced strain can cause an opening on the electronic band gap in individual carbon nanotubes [23,24,25,26]; however, we do not believe that this is the case here as the opening of the band gap would not cause as big a change as observed. Support for this assertion comes from our concurrent work on the effects of distortion on individual multi-walled carbon nanotubes (MWCNTs), which has shown that distorting the tube with pressure resulted in a maximum 1.5% increase in electrical resistance due to opening of the band gap [16]. Furthermore, changes in the band gap would result in a change to the contact type between the tungsten probes and the fiber, which is not observed here as demonstrated by the normalized I–V measurement. As may be seen from Figure 5, the shape of the I–V curves for measurements made with 0 nm, 100 nm and 200 nm probe displacement are essentially superimposable. Based upon our prior work [16], this suggests that there is no change in the band gap of the CNTs, which is certainly not sufficient to result in the 2 order of magnitude increase in electrical resistance observed with a probe displacement of 200 nm (cf., Figure 3).

**Figure 6:**
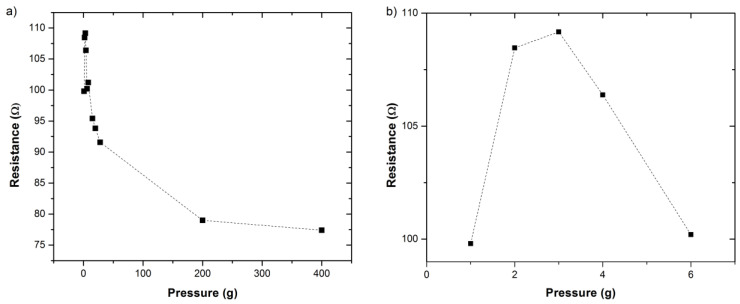
(**a**) Graph of resistance against pressure measured on the macro scale (see Section 2: Materials and Methods). (**b**) Expanded scale for the 1st four data points at the lowest pressure.

Another potential reason for the observed changes in resistance could be the formation of a quantum well under the tip, creating discrete energy states and therefore limiting current flow [27]. This again is unlikely on the scale used here, but can be confirmed if the same trend is also observed on the macro scale. Therefore, resistance measurements were also taken using macro scale probes to test for this hypothesis. Ideally, we would like to compare the nanoscale and macroscale measurements directly. However, we do not know the exact current path in the fiber, which drove us to perform this study to begin with, and it is unlikely that the current would run through the whole cross-section of the fiber, therefore electrical resistivity for comparison here is not a tangible metric. Therefore, we can only compare the trends between the two scales; Figure 6a shows the resistance calculated at 5 V against induced pressure depicted via the balance measurement in grams, with Figure 6b showing an expanded view of the lowest pressure values. As with the nanoscale measures, without knowing the contact area we cannot convert the measured weight into pressure or induced radial strain. As may be seen from Figure 6, there is an initial increase in electrical resistance as the applied pressure is increased while further pressure causes the resistance to decrease, with essentially little change above 200 g. We attribute the initial increase in the resistance to the same effects as that seen when taking the nanoscale measurements. The observed increase in resistance on both scales rules out the possibility that this is caused by a quantum well. The resistance range measured here would give a conductivity on the order of MS/m, which is in agreement with resistance measurements made by other groups on spun carbon nanotube fibers [11,12].

**Figure 7:**
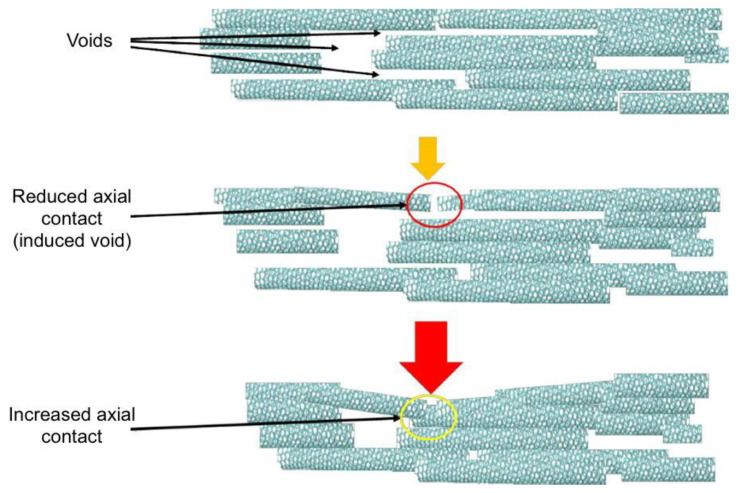
Illustrations depicting the effect of axial contact on void space between CNTs inside a fiber. Reduced contact can induce new voids, and increased contact can help close them.

Work carried out by Lekawa-Raus et al. investigated the piezoelectric properties of carbon nanotube fibers and showed that macroscale axial strain causes an increase in resistance measurements due to reduced radial CNT^…^CNT contact [28]. Here we have observed that initial radial strain causes similar results. One possible explanation would be that strain on the fiber structure causes axial CNT^…^CNT contact to decrease, which would indeed result in increased resistance [3,13]. Such a physical distortion would also result in an improved radial contact between individual CNTs with an increase in conductivity. We may have expected these effects to be minimal at this scale, as many of the tubes on the fiber’s surface will already be in radial contact and it would be thought that there is not enough displacement (maximum of 300 nm over a ~200 µm diameter fiber [29]) to close voids in the fiber; however, based upon the observation at both the nano- and macro-scales, we attribute this increase in resistance to a reduction in axial-contact between CNTs. The subsequent reduction in resistance with increased pressure observed at the macro scale (Figure 6a) can be attributed to the increased radial-contact between the nanotubes that on the macro scale is caused by closing of void spaces within the fiber. An illustration of the nature of void spaces as affected by axial contact is shown in Figure 7 below, to help visualize our hypothesis. Repeat measurements on a separate piece of fiber are shown in the Supplementary Materials, Figure S2. Figure 1c,d also shows compression of the fiber after radial strain is applied.

It is known that argon bombardment can damage CNTs, particularly when it comes to the π–π bonding [28], which is responsible for electron conduction in carbon nanotubes, and this damage could be observed using XPS with UPS [30]. In order to determine if the applied pressure might also cause similar damage, we have carried out XPS and UPS analysis on the pressed CNT fibers to determine if damage to the CNTs per se contributes to the increased resistance under radial pressure. Given that it is not possible to apply pressure in-situ during XPS analysis, we have instead chosen to study the effect of high pressure on a section of the fiber and compare it to a control fiber sample without any applied pressure via XPS. This pressure came in the form of 3 US-tons from a pneumatic press (see Section 2: Materials and Methods). XPS scans have been recorded for six areas each for the fiber sample with applied pressure as well as the control, including a survey scan and detailed scans of the C 1s, O 1s, Fe 2p, C Auger, and valence band with UPS. The high applied pressure on the fiber caused no observable change to the Auger peak, while the O 1s and Fe 2p peaks were found to vary across all six areas of each fiber (even in the absence of applied pressure), which is indicative of the inconsistency in the fiber, and therefore cannot be used to gain any information other than that iron and oxygen are both present in varying quantities throughout the fiber.

**Figure 8:**
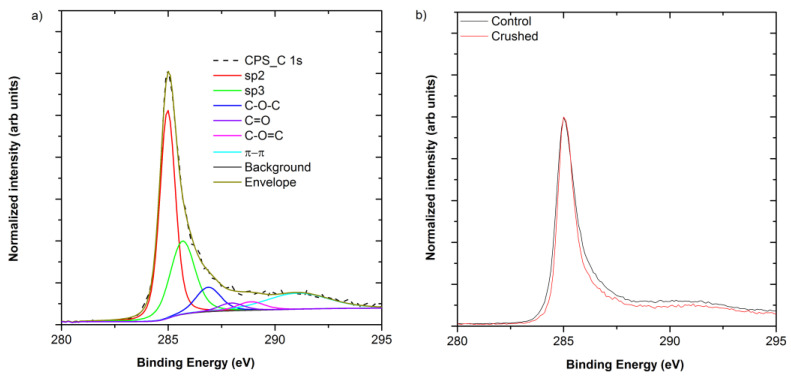
(**a**) Normalized averaged C 1s peaks of control and crushed fiber. (**b**) Insert shows the fit applied to the C 1s spectrum of the control sample.

**Figure 9:**
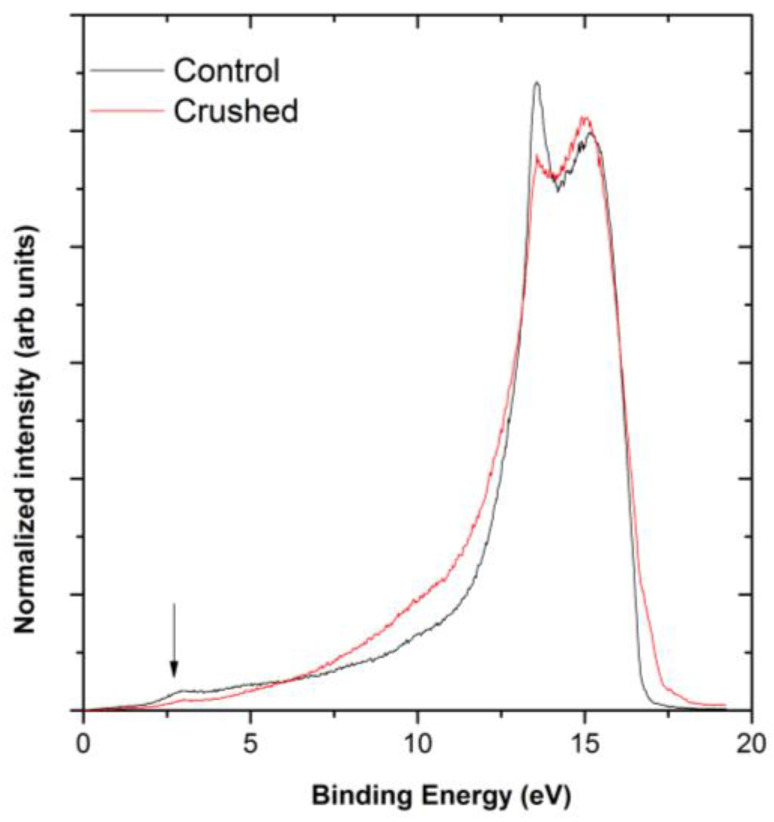
Normalized UV photoelectron spectroscopy (UPS) valence band scans of control and crushed fiber with arrow indicting π-orbital.

Figure 8a shows the averaged normalized C 1s peaks of the control and crushed CNT fiber. The C 1s peak (Figure 8b) may be fitted with six peaks. Those centered at 285.0 eV and 285.7 eV are assigned to sp^2^ and sp^3^ carbon, respectively, while those at 286.9 eV, 288.0 eV and 288.9 eV are representative of C-O-C, C=O and O-C=O bonds associated with the oxygen content of CNTs [20,31]. Finally, the broad peak 291.1 eV corresponds to the π–π* component [32]. It should be noted that the π–π* peak could be further split into multiple components, but is not necessary for the analysis carried out here. From Figure 9, it may be seen that the contribution from the π–π* component has reduced after crushing, and analysis of the components shows that the π–π* fraction has reduced from 16.1 ± 0.9% to 10.14 ± 0.9% of the total C 1s, which is consistent with the observed reduction in electrical conductivity.

The UPS was carried out using the He I emission and shows that crushing the fiber did not cause a shift in the VB band, suggesting no shift in the band gap. The Fermi edge continued to have a crossing at the zero and secondary electron cut offs indicating the work function, which is the small shoulder on the secondary electron cut off and remained at 4.4 ± 0.1 eV in Figure 9, which agrees with the published literature and is attributed to contamination [16]. This also corroborates the observations from the nanoscale measurements, as seen in Figure 5, which showed that the contact type has not changed. However, after the fiber was crushed, the peaks on the valence band scan are reduced. These peaks correspond to the orbitals under examination, including the π-orbitals which are marked with an arrow [32,33], and indicate that they have been suppressed by the increased pressure. This would typically cause an increase in resistance; however, these data do not directly provide a measure of the overall change in the resistance of the fiber.

## 4. Conclusions

Carbon nanotube fibers have the potential to replace copper wiring for electrical current transport; however, in use, these fibers may be put under radial strain on both the nano- and macro-scales. It is known that axial strain increases the electrical resistance of CNT fibers, and we have shown here that radial strain can also increase the resistance. We attribute this mainly to the reduction in axial contact between nanotubes in the fiber; however, there is also a plausible contribution to the increased resistance caused by damage to the π–π bonding of the nanotubes when under pressure. We have also observed that, on the macro scale, after an initial increase in resistance with pressure, the resistance reduces with further pressure, which we attribute to increased radial CNT contacts as voids in the fiber are closed. These results show that care must be taken in putting CNT fibers under radial strain so that it is not in the regime of higher resistance, be it in the form of irregular crimping at contacts or otherwise. We are working on methods to obtain a more consistent fiber composition, which will allow us to better study the effect of strain on electrical resistance at the nanoscale.

## Data Availability

The data presented in this study are available on request from the corresponding author.

## Supplementary Materials

The following are available online at https://www.mdpi.com/article/10.3390/ma14092106/s1, Figure S1: Graph of electrical resistance of a CNT fiber measured using 20 µm probe separation against STM probe displacement: black squares show probe approach and red circles showing retraction, Figure S2: Graph of repeat resistance against pressure measured on the macro scale.
