# Supplementary material for: Effect of Applied Pressure on the Electrical Resistance of Carbon Nanotube Fibers
Source: Materials (Basel). 2021 Apr 21;14(9):2106. doi: 10.3390/ma14092106 (PMC8122425; doi:10.3390/ma14092106)
Supplement: Supplementary file 1 [file materials-14-02106-s001.zip › materials-1134134-supplementary.pdf]

Effect of Applied Pressure on the Electrical Resistance of Carbon Nanotube Fibers

Chris J. Barnett, James D. McGettrick, Varun Shenoy Gangoli, Ewa Kazimierska, Alvin Orbaek White and Andrew R. Barron

Supplemental Information

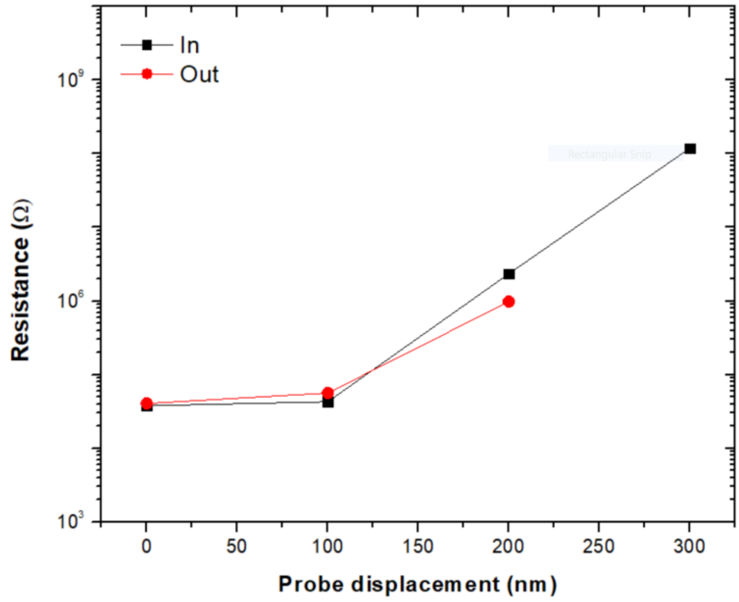

Figure S1. Graph of electrical resistance of a CNT fiber measured using 20 μm probe separation against STM probe displacement: black squares show probe approach and red circles showing retraction.

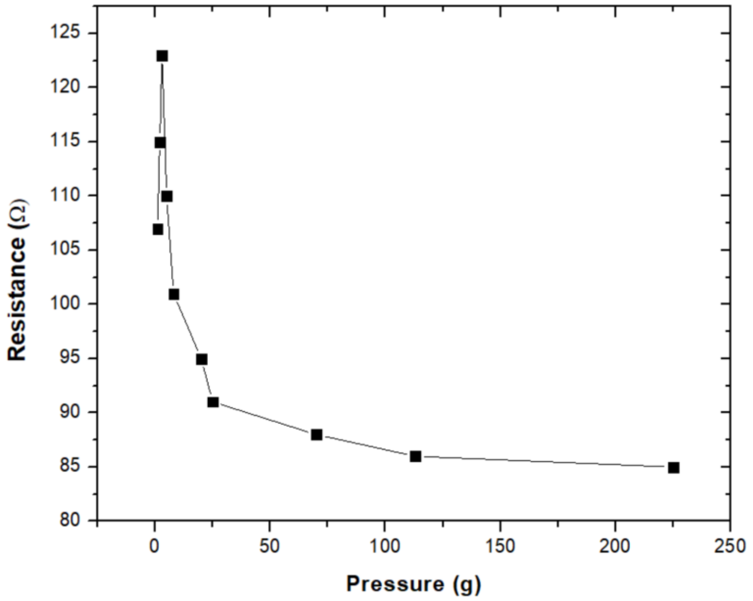

Figure S2. Graph of repeat resistance against pressure measured on the macro scale.
